# Reduced T-cell Numbers and Elevated Levels of Immunomodulatory Cytokines in Metastatic Prostate Cancer Patients De Novo Resistant to Abiraterone and/or Enzalutamide Therapy

**DOI:** 10.3390/ijms20081831

**Published:** 2019-04-13

**Authors:** Sumanta K. Pal, Dayson Moreira, Haejung Won, Seok Woon White, Pryanka Duttagupta, Marc Lucia, Jeremy Jones, JoAnn Hsu, Marcin Kortylewski

**Affiliations:** 1 Medical Oncology and Experimental Therapeutics; City of Hope Comprehensive Cancer Center, Duarte, CA 91010, USA; jjones@coh.org (J.J.); johsu@coh.org (J.H.); 2 Department of Immuno-Oncology, Beckman Research Institute at City of Hope Comprehensive Cancer Center, Duarte, CA 91010, USA; dmoreira@coh.org (D.M.); haejungw@gmail.com (H.W.); sewhite@coh.org (S.W.W.); pduttagupta33@gmail.com (P.D.); marclp@stanford.edu (M.L.)

**Keywords:** castration-resistant prostate cancer, abiraterone, enzalutamide, CRPC, cytokines, ADT

## Abstract

Currently, there are two Food and Drug Administration (FDA)-approved drugs for androgen deprivation therapy (ADT) of metastatic castration-resistant prostate cancer (mCRPC) patients: abiraterone and enzalutamide. However, our understanding of the effect of these therapies on the immune system in mCRPC patients remains limited. Here, we examined how abiraterone and enzalutamide treatment affects levels of soluble immune mediators in plasma and in circulating immune cells of 44 mCRPC patients. We found that the baseline levels of cytokines fibroblast growth factor (FGF), granulocyte-macrophage colony-stimulating factor (GM-CSF), interleukin 10 (IL-10), and IL-6 were significantly lower in ADT-sensitive compared to de novo resistant patients. In addition, resistant patients showed significantly lower T cell frequencies. When comparing the levels of cytokines over the course of treatment, we observed that the levels of proinflammatory mediators, such as interferon-γ (IFN-γ), IL-5, macrophage inflammatory protein 1 alpha (MIP-1α), and tumor necrosis factor alpha (TNFα), were significantly increased in the ADT-sensitive patients. At the same time, the abiraterone/enzalutamide therapy did not reduce the percentage of tolerogenic myeloid cell populations, such as polymorphonuclear myeloid-derived suppressor cells, which retained unaltered expression of programmed death-ligand 1 (PD-L1) and B7-H3. Overall, our results suggest that certain immune markers, such as IL-6 and the frequency of effector T cells, could be predictive of therapeutic response to ADT therapies in mCRPC patients.

## 1. Introduction

Androgen deprivation therapy (ADT) is the standard treatment for advanced stages of localized and/or metastatic prostate cancers [1]. Although there is a dramatic initial response corresponding with a significant decrease of prostate-specific antigen (PSA) levels and objective response in the metastatic sites, the majority of the patients will eventually develop resistance to ADT and progress towards metastatic castration-resistant prostate cancer (mCRPC) [2,3,4]. There are several mechanisms, which center on the regulation of androgen receptor (AR) signaling, of ADT resistance that can contribute to the prostate cancer progression. These mechanisms include AR mutations leading to amplification and hypersensitivity, mutations in coactivators/corepressors, and alternative androgen production, such as androgen biosynthesis in the tumor microenvironment, androgen-independent AR activation, and alternative oncogenic signaling [1,4].

Abiraterone acetate (Zytiga) and enzalutamide (Xtandi) are two currently Food and Drug Administration (FDA)-approved drugs for the treatment of newly diagnosed metastatic prostate cancer patients who are still sensitive to the manipulation of androgen signaling [1,5]. Both drugs target AR signaling, but use different mechanisms of action. Abiraterone inhibits the enzyme CYP17A1 involved in the synthesis of androgens in organs like adrenal glands, testes, and the prostate tumor itself [1]. In contrast, enzalutamide binds directly to the AR, and this binding prevents nuclear translocation and coactivation recruitment of the ligand-receptor complex [5]. Despite the increasing clinical application of both drugs, there is little consensus regarding the optimal sequence of their application in treating mCRPC patients. Currently, treatment selection hinges on clinical parameters, such as the presence or absence of symptomatic disease and the distribution of metastases [6]. A biomarker-based selection strategy remains elusive. To this end, we had recently performed a prospective assessment of patients receiving abiraterone and enzalutamide for mCRPC [7]. Our initial efforts focused on the capture of circulating tumor cells (CTCs) with single-cell RNA-seq assessment for differentially regulated genes in patients sensitive or resistant to the treatment [7]. This work identified transforming growth factor-β (TGF-β) and cyclin D1 (CCND1) as putative mediators of resistance as confirmed in preclinical models of prostate cancer [8].

Beyond the previously identified pathways, recent evidence points to the inflammatory signaling as a key driver of prostate cancer growth and metastasis [9]. Emerging evidence suggests that inflammation-related markers may change during the course of therapy with abiraterone [10]. Fan et al. showed that the immune inflammation index (platelet multiplied by neutrophil divided by lymphocyte numbers) could be a significant predictor of the overall survival [10]. In another study, Zhao and colleagues recently reported an assessment of over 9000 prostatectomy specimens, linking programmed death-ligand 2 (PD-L2) to poorer biochemical recurrence-free survival and prostate cancer-specific survival. However, these tissue samples were collected from early-stage prostate cancer patients, and there was a limited capability to interpret the utility of this biomarker in the context of mCRPC [11]. Interestingly, the immunomodulatory effects of enzalutamide were documented in mouse prostate cancer models, such as transgenic adenocarcinoma mouse prostate C2 (TRAMP-C2) [12]. While the enzalutamide alone did not affect CD4^+^ T-cell proliferation or regulatory T-cell function, the combination of enzalutamide with Twist-vaccine improved the efficacy of the treatment resulting in increased mouse survival with advanced prostate cancer [12].

Overall, there is emerging evidence to suggest that treatments with either enzalutamide or abiraterone could have immunomodulatory effects in patients with advanced prostate cancers. Therefore, we decided to evaluate changes in immune mediators in blood specimens from mCRPC patients who were either sensitive or resistant to the abiraterone or enzalutamide treatment. 

## 2. Results

### 2.1. Patient Characteristics

A total of 44 patients were enrolled between April 2014 and July 2017. The median age of the cohort was 74 years (55–94 years range), and the median baseline PSA was 63.63 ± 143.77 ng/mL. Eleven patients received abiraterone, and 23 patients received enzalutamide. In addition, there were five patients receiving abiraterone that had received prior enzalutamide, and seven patients receiving enzalutamide had received prior abiraterone. No patients discontinued therapy on account of toxicity, and all had investigator-defined progression, which was either based on biochemical, radiographic, or symptomatic progression. Maximal PSA decline of 30% or more was achieved in 25 patients, while a maximal PSA decline of 50% or more was achieved in 19 patients. Seven patients had no PSA response, while six patients only had the PSA baseline levels analyzed. The median duration of treatment (DOT) for the overall cohort was 17 months (range, 1–56). No significant difference was noted in the median DOT for patients receiving abiraterone (23 months) or enzalutamide (22 months). Patients were divided into two groups based on median DOT. The two groups were denoted as resistant where patients had a more limited DOT (median 4.75 months; range, 1–12 months) and sensitive where patients had a prolonged DOT (median 34.8 months; range, 12–56 months). Nine out of 44 patients remain under treatment in January 2019.

### 2.2. mCRPC Patients Sensitive and Resistant to Abiraterone/Enzalutamide Therapy Show Different Pattern of Plasma Cytokines and Chemokines before Treatment Initiation

**Figure 1:**
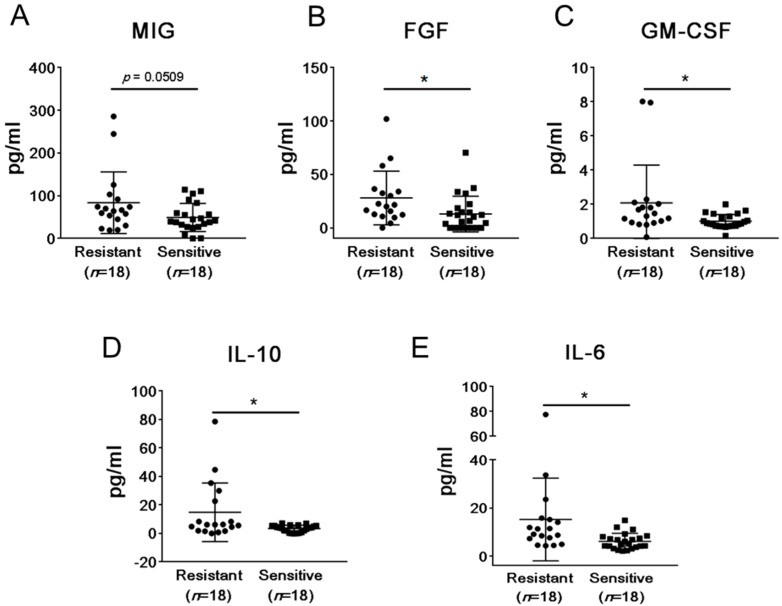
The comparison of plasma levels of immune-related protein mediators between patients sensitive and resistant to abiraterone and/or enzalutamide treatment. Baseline levels of 30 soluble immune mediators were analyzed in plasma samples derived from 23 patients responding to therapy (sensitive) and 18 patients resistant to therapy with progressing disease using Luminex analysis. Shown are results for the significantly altered analytes: monokine induced by gamma interferon (MIG) (**A**), fibroblast growth factor (FGF) (**B**), granulocyte-macrophage colony-stimulating factor (GM-CSF) (**C**), interleukin 10 (IL-10) (**D**), and IL-6 (**E**). * *p* < 0.05.

Among the 44 mCRPC patients undergoing abiraterone and/or enzalutamide therapy included in our study, 23 patients showed durable responses, while tumors recurred in 18 patients as assessed by rising PSA values. We first assessed whether both groups of patients showed differences in baseline plasma levels of soluble immune mediators. Levels of 30 cytokines, chemokines, and growth factors (see Materials and Methods section and Supplementary Table S1) were measured in plasma samples collected from patients before treatment initiation. We found a significant difference (*p* < 0.05) in the levels of fibroblast growth factor (FGF), granulocyte-macrophage colony-stimulating factor (GM-CSF), interleukin 6 (IL-6), and IL-10 when comparing patients de novo resistant and sensitive to abiraterone and/or enzalutamide therapy (Figure 1). None of the chemokines examined showed any significant difference in their baseline levels although monokine induced by gamma interferon/C-X-C motif chemokine ligand 9 (MIG)/CXCL9 (interferon-γ (IFNγ)-induced chemokine), recently reported to have T cell inhibitory effects in prostate cancer [13], showed higher average baseline levels in the treatment-resistant patients at 83.81 pg/mL compared to the treatment-sensitive group at 49.09 pg/mL (*p* = 0.051, Figure 1A). In the abiraterone/enzalutamide-resistant patients, baseline levels of FGF, GM-CSF, IL-6, and IL-10 were significantly higher compared to the treatment-sensitive patients. The average baseline level of FGF in the resistant and sensitive group was 27.80 pg/mL and 12.94 pg/mL, respectively (*p* = 0.0251, Figure 1A). For GM-CSF, the average baseline level in the resistant group was 2.09 pg/mL, and in the sensitive group, the average baseline level was 1.01 pg/mL (*p* = 0.0284, Figure 1B). For IL-10, the average baseline level in the resistant group was 14.88 pg/mL, and in the sensitive group, the average baseline level was 3.38 pg/mL (*p* = 0.011, Figure 1C). For IL-6, the average baseline level in the resistant group was 15.22 pg/mL, and for the sensitive group, the average baseline level was 6.13 pg/mL (*p* = 0.0176, Figure 1E). Interestingly, IL-6 levels were detectable but not significantly elevated in patients with more than five CTC/7.5 mL of blood when compared to patients with less than five CTC/7.5 mL of blood (Supplemental Figure S1). Taken together, the analysis of plasma levels of soluble immune mediators suggests that de novo resistance to abiraterone and/or enzalutamide therapy may be related to systemic effects of elevated levels of potentially tumorigenic and immunosuppressive mediators in patients’ plasma. 

### 2.3. Abiraterone/Enzalutamide Therapy Does Not Affect Expression of Programmed Death-Ligand 1 (PD-L1) and B7H3 Immune Checkpoint Regulators on Circulating Myeloid Suppressor Cells in mCRPC Patients

**Figure 2:**
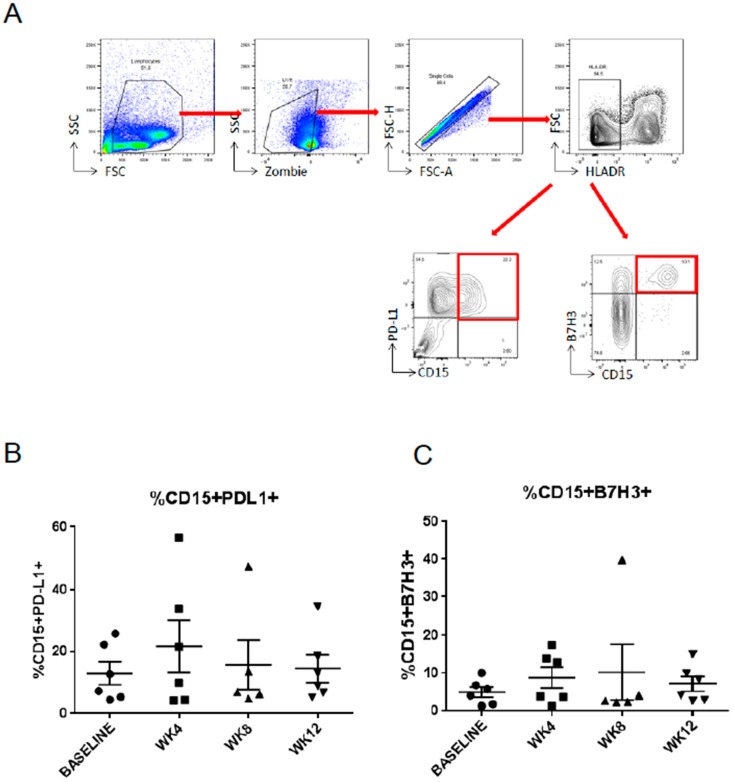
The expression of programmed death-ligand 1 (PD-L1) and B7-H3 on polymorphonuclear myeloid-derived suppressor cells (PMN-MDSCs) derived from prostate cancer patients. (**A**) Gating strategy to assess the expression of PD-L1 and B7-H3 on HLA-DR^−^ CD15^+^ MDSCs in peripheral blood mononuclear cell (PBMC) using flow cytometry. Shown are flow cytometry dot plots with red arrows indicating the gating strategies and red rectangles the population of interest. (**B**) The surface expression of PD-L1 and (**C**) B7-H3 on the HLA-DR^−^ CD15^+^ MDSCs as measured during the course of abiraterone and/or enzalutamide treatment (*n* = 6 patients). WK: Week; SSC: Side Scatter; FSC: Forward scatter; HLADR: Human Leukocyte Antigen—DR isotype.

The treatment with enzalutamide can promote immunosuppression in the prostate tumor microenvironment by increasing immune checkpoint regulators like PD-L1 in immune cells, such as dendritic cells (DCs) [14]. We recently identified a subpopulation of polymorphonuclear myeloid-derived suppressor cells (PMN-MDSCs) as a major type of tolerogenic immune cells accumulating in the blood of prostate cancer patients with progression of the disease [15]. Thus, we examined changes in the expression of two immune checkpoint regulators, namely PD-L1 and B7-H3, in PMN-MDSCs during the course of abiraterone/enzalutamide treatment (Figure 2A). The surface expression of PD-L1 was compared between blood samples collected over the course of treatment with abiraterone/enzalutamide therapy at week 4, 8, and 12. We found PD-L1 and B7-H3 expression on approximately 10% and 5% of PMN-MDSCs, respectively. However, there were no significant changes in the level of PD-L1 (Figure 2B) or B7-H3 (Figure 2C) over the time of treatment. In addition, we did not observe differences in the percentage of PD-L1- and B7-H3-positive PMN-MDSCs between the groups. Thus, our results failed to detect any significant effects of abiraterone/enzalutamide therapy on the expression of PD-L1 and B7-H3 immune checkpoints on tolerogenic MDSCs in mCRPC patients.

### 2.4. Modulation of Plasma Levels of Soluble Immune Mediators during Abiraterone/Enzalutamide Therapy in mCRPC Patients

**Figure 3:**
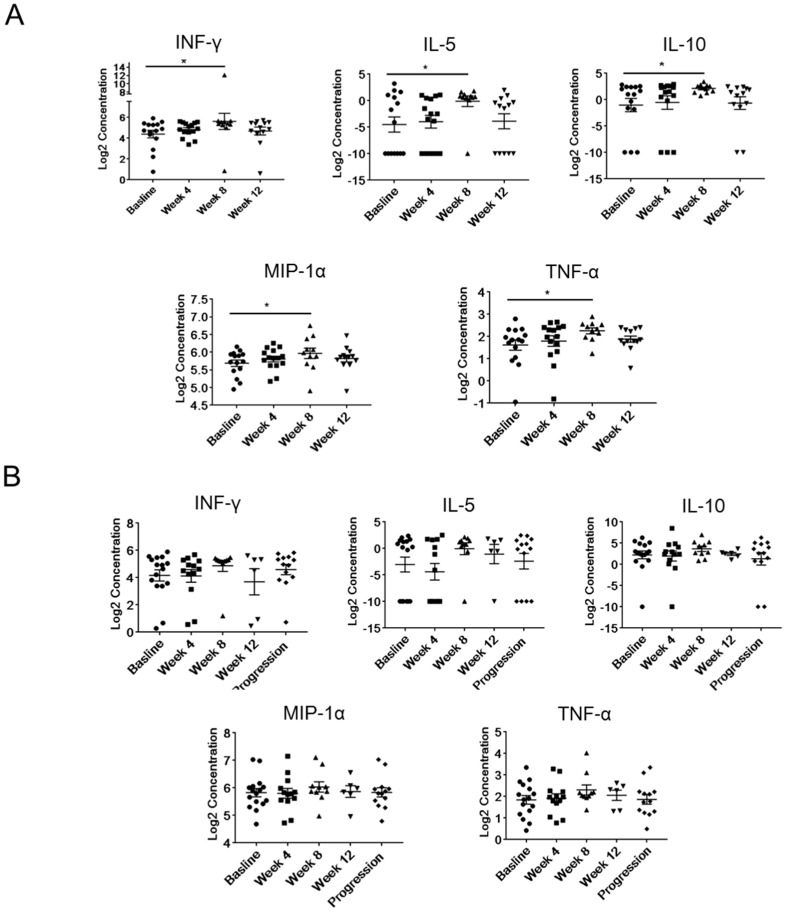
The longitudinal analysis of plasma levels of immune-related mediators in mCRPC patients undergoing abiraterone and/or enzalutamide treatment. Longitudinal changes of plasma level of IFNγ, IL-5, IL-10, MIP-1α, and TNFα were measured using Luminex assay. Shown are results derived from treatment-sensitive (**A**) (Baseline, *n* = 15; week 4, *n* = 15; week 8, *n* = 11; week 12, *n* = 13) or treatment-resistant patients (**B**) (Baseline, *n* = 16; week 4, *n* = 13; week 8, *n* = 10; week 12, *n* = 6; progression, *n* = 13). * *p* < 0.05.

Next, we examined whether abiraterone/enzalutamide therapy correlates with any systemic immunomodulatory effects within the initial 12 weeks of treatment by measuring the plasma level of 30 cytokines, chemokines, and growth factors from samples collected before start of treatment (at the baseline), and during treatment at weeks 4, 8, and 12 from the sensitive (Figure 3A) or the resistant patients (Figure 3B). In treatment-sensitive patients, we found that the plasma levels of interferon-γ (IFNγ), IL-5, IL-10, and tumor necrosis factor alpha (TNFα) and the chemokine macrophage inflammatory protein 1 alpha (MIP-1α)/chemokine (C-C motif) ligand 3 (CCL3) were significantly increased at week 8 of therapy compared to the baseline but not at week 12 (Figure 3A). However, there were no changes in the levels of IFNγ, IL-5, IL-10, MIP-1α, and TNFα even after the disease progressed in resistant patients (Figure 3B). The elevated levels of proinflammatory mediators, such as IFNγ, MIP-1α, and TNFα, together with T helper 2 (Th2)-promoting interleukins suggest potential activation of T-cell mediated immune responses in mCRPC patients responding to abiraterone/enzalutamide therapy.

### 2.5. Differences in Baseline Frequencies of T Cells between Treatment Resistant and Sensitive Patients

**Figure 4:**
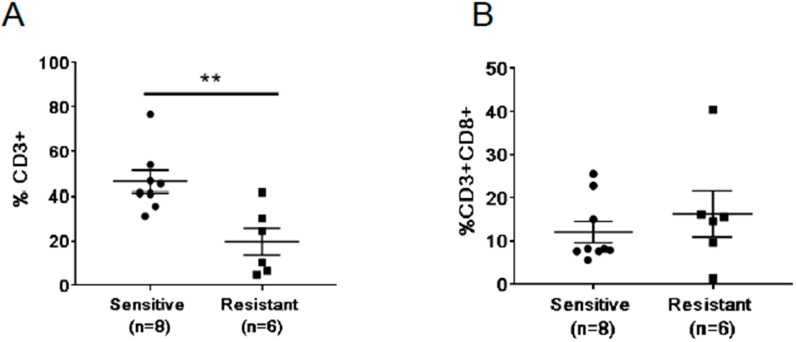
Differences in frequencies of circulating T cells between abiraterone and/or enzalutamide treatment-resistant and -sensitive mCRPC patients. The percentage of CD3+ (**A**) and CD3+CD8+ (**B**) T cells in the peripheral blood were analyzed using flow cytometry. ** *p* < 0.01.

The observation of increased T cell-regulating cytokines, IL-5 and IL-10, in the sensitive patients prompted us to assess numbers of T cells in the peripheral blood (Figure 4). Due to limited cell numbers, we have analyzed a total of eight sensitive and six resistant patients. We found that the average percentage of CD3^+^ cells in the sensitive group at 46.44% was significantly higher compared to the resistant group at 19.71% (*p* = 0.0048; Figure 4A). However, the percentage of CD3^+^CD8^+^ cytotoxic T cells in the sensitive group was not significantly different compared to the resistant group (*p* = 0.4363, Figure 4B). We also did not find any changes in the expression of PD1 in the CD8^+^ T cells over the time of the treatment (Supplemental Figure S2). This data suggests that the reduced percentage of T cells at baseline could be related to the outcome of abiraterone/enzalutamide therapy. 

## 3. Discussion

Our analysis of plasma levels of immune mediators revealed four factors, namely FGF, GM-CSF, IL-6, and IL-10, that were consistently and significantly elevated in the circulation of mCRPC patients resistant to abiraterone and/or enzalutamide. In contrast, only the treatment sensitive and not resistant patients showed an increase of proinflammatory mediators, such as IFN-γ, IL-5, IL-10, MP-1α, and TNFα, during treatment. Consistent with the increased levels of immunoregulators, the frequencies of T cells in circulation prior to the treatment were significantly lower in de novo resistant patients compared to sensitive patients. These results suggest that potently immunosuppressive tumor microenvironment may limit the outcome of ADT therapies. At the same time, there was no change detected in the immune checkpoint regulators, PD-L1 and B7-H3, in patients’ HLA-DR^−^CD15^+^ MDSCs. These results suggest the need for more extensive studies to assess the potential of using immune-related plasma markers to assess the potential outcome of abiraterone and/or enzalutamide treatment. 

FGF, GM-CSF, IL-6, and IL-10 are growth factors and cytokines well known for playing important roles in promoting tumor progression and/or immune evasion. FGF supports tumor cell propagation, angiogenesis, and metastasis in various types of solid cancers [16,17]. In addition, elevated FGF correlated with the increase of M2 macrophages in the tumor microenvironment [18,19]. GM-CSF is primarily involved in the regulation of myeloid cell differentiation, maturation, and function [20]. However, recent studies suggest that GM-CSF does have immune-suppressive roles in the tumor microenvironment, such as supporting the expansion of potently immunosuppressive MDSCs [21,22]. While we did not observe changes in the percentage of PMN-MDSCs among tested samples, our current studies confirmed the presence of these immunosuppressive cells in the circulation of late-stage mCRPC patients as we previously reported [15]. Moreover, IL-10 is a key immunoregulatory mediator which suppresses T cell functions and induces regulatory T cell development, thereby promoting tumor immune evasion [23,24,25]. This might also contribute to the lower frequency of T cells observed in patients before treatment initiation. 

Finally, IL-6 is a pro-inflammatory cytokine with a well-established role in promoting androgen-independent phenotype of prostate cancer cells as well as immunosuppression in the prostate tumor microenvironment, which can be induced by a plethora of danger signals [26,27]. In cancer patients, IL-6 induces development and immunosuppressive function of MDSCs and tumor-associated macrophages through signal transducer and activator of transcription 3 (STAT3)-mediated mechanism [15,26,27,28,29]. Using the same cohort of patients, we previously showed that a baseline CTC count of over five is associated with more rapid disease progression and that the increase of CTCs correlates with the emergence of drug resistance [30]. Intriguingly, patients with an increased percentage of CTCs showed increased levels of plasma IL-6 although, due to a limited number of patients, this correlation was not significant (Supplemental Figure S1). 

Over the course of treatment, there was an increase in the cytokine levels of IFN-γ, IL-10, IL-5, MIP-1α, and TNF-α at week 8 when compared to the baseline. Interestingly, there were no significant changes in any soluble mediators tested during the therapy in resistant group. IFN-γ and TNF-α play important roles in cytotoxic T cell functions and anti-tumor activity [31,32]. MIP-1α (CCL3) is a chemo-attractant for CD8^+^ T cells and stimulates CD8^+^ effector functions in viral infection and anti-tumor activity in cancers by activating DCs and natural killer cells [33,34]. In addition, we also observed a transient but significant increase of Th2 cytokine IL-5 and IL-10 in the sensitive group, likely related to the T cell activation. Ardiani et al. demonstrated that abiraterone/enzalutamide treatment sensitized tumor cells to T cell-mediating killing in a mouse model of mCRPC [8]. Others have shown that ADT, and specifically enzalutamide and flutamide, can impair the adaptive immune response by interfering with T cell priming [35]. In addition, a recent study in a mouse prostate tumor model found that enzalutamide did not induce immune activation as measured by lack of CD4^+^ and CD8^+^ proliferation [12]. These preclinical observations seem to correlate with the lack of changes in T cell percentage and activation during the course of abiraterone/enzalutamide treatment in our study. However, the higher percentage of T cells detectable before treatment in patients’ plasma was associated with better response to ADT treatment in our study. Consistently, our data provide evidence that a higher level of circulating T cells and increased production of T cell activation-related cytokines during treatment may indicate favorable prognosis. 

Bishop and colleagues showed a significant increase in blood circulating PD-L1/2^+^ DCs in patients with progressing mCRPC treated with enzalutamide when compared to patients responding to enzalutamide treatment or prior to the start of treatment [14]. However, the number of patients examined in this study was relatively small (eight responding vs. four non-responding patients). While our study did not assess the expression of PD-L1/2 on DCs, we identified PD-L1 expression on the major myeloid suppressor population, PMN-MDSCs, in mCRPC patients’ circulation. However, we did not observe any difference between the groups or changes in the expression of PD-L1 or B7-H3 during the course of treatment. We also did not find any changes in the expression of PD1 in the T CD8^+^ cells in the circulation of sensitive or resistant patients during the treatment. Taken together, our results suggest that the mechanism of de novo resistance to abiraterone/enzalutamide treatment in patients with metastatic prostate cancers can be related to potently immunosuppressive tumor microenvironment. Our studies indicate that immune markers, such as IL-6 levels or the frequency of effector T cells, may be predictive of the outcome of treatment with ADT therapies in patients with metastatic prostate cancers. These observations need to be validated in further clinical studies on a larger population of metastatic prostate cancer patients. 

## 4. Materials and Methods

### 4.1. Patient Selection and Clinical Assessment

Patients eligible for this clinical study were cytologically or pathologically diagnosed with prostate cancer and had radiographic evidence of metastatic disease. Patients were defined by the clinician as having mCRPC, based most commonly on (1) castrate levels of testosterone (<50 ng/dL) and (2) failure of initial hormonal blockade. With appropriate counseling regarding treatment options, subjects must have opted to receive either abiraterone or enzalutamide. The patients must also have had an anticipated monitoring plan, including serial monthly collections of prostate-specific antigen (PSA); consent was obtained to pair these collections with blood collection for correlative studies under the Institutional Review Board (IRB) approved clinical protocol (IRB#13141). Duration of treatment (DOT) was characterized for all patients, representing the time elapsed from the start of treatment with abiraterone or enzalutamide to withdrawal of treatment. Change in PSA was also computed for the cohort relative to baseline values. The protocol and the relevant informed consent form were approved (IRB#13141) by the institutional scientific review committee, data safety monitoring board, and the institutional review board at the City of Hope. All patients enrolled provided written informed consent, and the study was conducted in accordance with the amended Declaration of Helsinki and the International Conference on Harmonization Guidelines.

### 4.2. Blood Processing, Plasma, and Peripheral Blood Mononuclear Cell (PBMC) Preparation

Patients’ blood was collected, processed, and stored as described previously [36]. Briefly, plasma and PBMCs were separated using centrifugation at 1500× *g* for 10 min. Plasma samples were stored at −80 °C, and PBMCs were resuspended (1:1) in Cryostor CS5 media (BioLife Solutions, Bothell, WA, USA) and transferred to liquid nitrogen within 48 h after freezing at −80 °C. 

### 4.3. Plasma Biomarker Assessment

Patients’ plasma samples were analyzed for 30 soluble immune mediators using Luminex analysis as described previously [36]. The 30 soluble immune mediators that were examined were epithelial growth factor (EGF), eotaxin, FGF-basic, granulocyte-colony stimulating factor (G-CSF), GM-CSF, hepatocyte growth factor (HGF), interferon-α, interferon-γ, IL-10, IL-12, IL-13, IL-15, IL-17, IL-1RA, IL-1B, IL-2, IL-2R, IL-4, IL-5, IL-6, IL-7, IL-8, IP-10, MCP-1, MIG, MIP-1α, MIP-1β, regulated on activation, normal T cell expressed and secreted (RANTES), TNFα, and vascular endothelial growth factor VEGF. 

### 4.4. Flow Cytometry

Frozen PBMCs were quickly thawed at 37 °C in a water bath. Once thawed, cells were washed with PBS and incubated in RPMI media (ThermoFisher Scientific, Waltham, MA, USA) supplemented with 20% matched plasma for 2 h in a 37 °C incubator. Cells were then washed in PBS supplemented with DNase (Roche, Basel, Switzerland) and pre-incubated with CD16/32 antibody to block non-specific binding. Cells were stained with UV-Zombie viability marker (Biolegend, San Diego, CA, USA) followed by staining with fluorescence-labeled antibodies, anti-human CD3, CD4, CD8, PD1 antibodies (Thermo Fisher Scientific, Waltham, MA, USA). For intracellular staining of FoxP3, cells were fixed and permeabilized after surface staining using Anti-Human FOXP3 Staining Set according to the manufacturer’s protocol (Thermo-Fisher Scientific, Waltham, MA, USA). Flow cytometry data were collected on the BD Fortessa (BD Biosciences, San Diego, CA, USA), and the acquired data were analyzed using FlowJo software (version 10.4.1, TreeStar, Woodburn, OR, USA). 

### 4.5. Statistics

Unpaired student *t*-tests and two-way ANOVA followed by Bonferroni post-tests were used for the statistical analysis; *p* < 0.05 was considered significant. All statistical analyses were computed using GraphPad Prism v7.0. 

## Supplementary Materials

Supplementary materials can be found at https://www.mdpi.com/1422-0067/20/8/1831/s1.

## Abbreviations

ADT	androgen deprivation therapy
AR	Androgen receptor
CTCs	circulating tumor cells
DCs	dendritic cells
DOT	duration of therapy
FGF	fibroblast growth factor
GM-CSF	granulocyte-macrophage colony-stimulating factor
mCRPC	metastatic castrate-resistant prostate cancer
PBMC	peripheral blood mononuclear cell
PD-L1	programmed cell death ligand 1
PMN-MDSCs	polymorphonuclear myeloid-derived suppressor cells
PSA	prostate-specific antigen
