# Supplementary figures and images for: Reduced T-cell Numbers and Elevated Levels of Immunomodulatory Cytokines in Metastatic Prostate Cancer Patients De Novo Resistant to Abiraterone and/or Enzalutamide Therapy
Source: Int J Mol Sci. 2019 Apr 13;20(8):1831. doi: 10.3390/ijms20081831 (PMC6515443; doi:10.3390/ijms20081831)

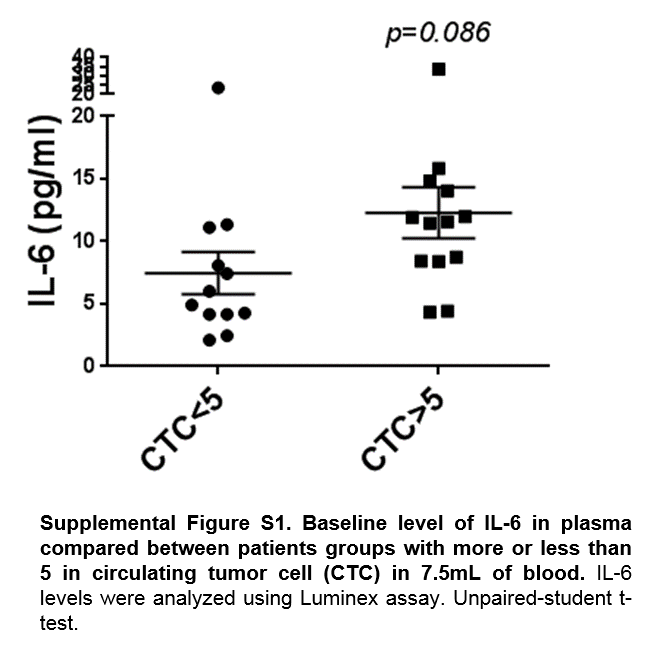

Supplement: Supplementary file 1 [file ijms-20-01831-s001.zip › ijms-482331 Suppl/Supplemental Figure 1.tif]

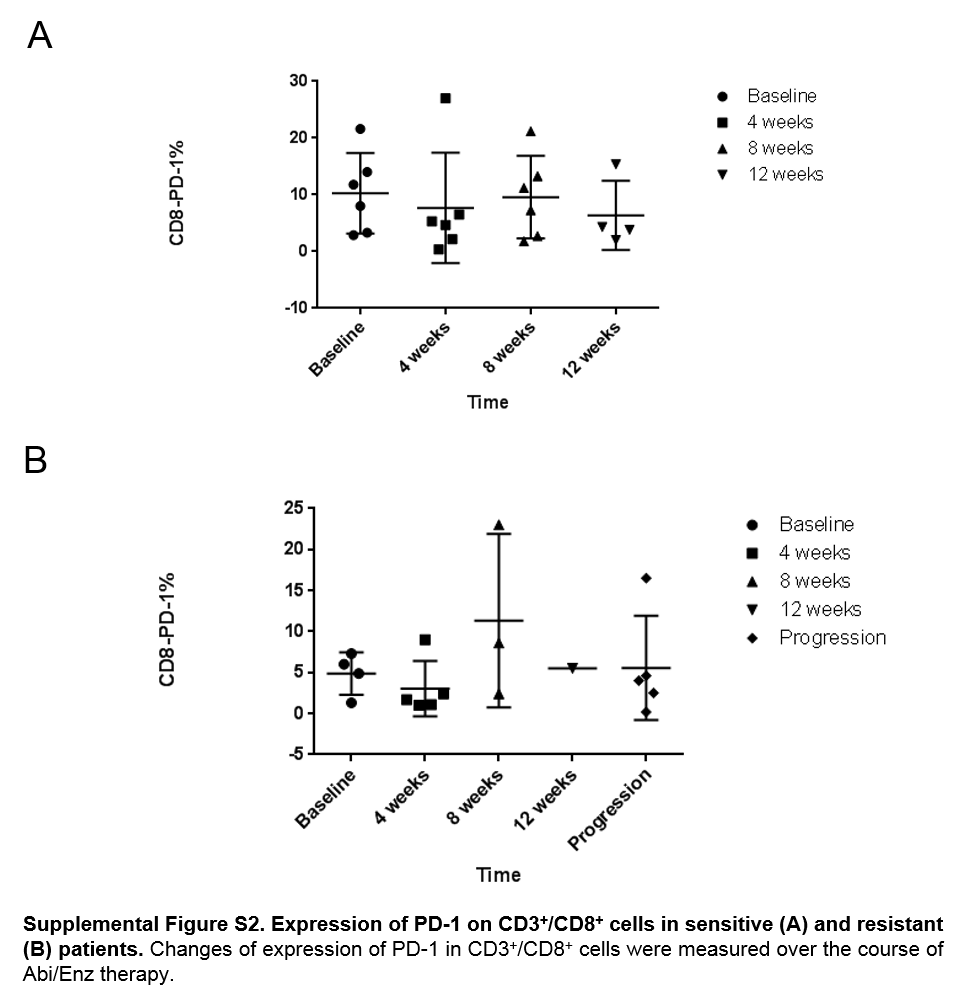

Supplement: Supplementary file 1 [file ijms-20-01831-s001.zip › ijms-482331 Suppl/Supplemental Figure 2.tif]
